# A Review of the Advances and Challenges in Measuring the Thermal Conductivity of Nanofluids

**DOI:** 10.3390/nano12152526

**Published:** 2022-07-22

**Authors:** Reinaldo R. Souza, Vera Faustino, Inês M. Gonçalves, Ana S. Moita, Manuel Bañobre-López, Rui Lima

**Affiliations:** 1 Metrics, Mechanical Engineering Department, University of Minho, Campus de Azurém, 4800-058 Guimarães, Portugal; vera_f_87@hotmail.com (V.F.); inesmaiag@gmail.com (I.M.G.); rl@dem.uminho.pt (R.L.); 2 Advanced (Magnetic) Theranostic Nanostructures Lab, International Iberian Nanotechnology Laboratory, 4715-330 Braga, Portugal; manuel.banobre@inl.int; 3 IN+, Center for Innovation, Technology and Policy Research, Instituto Superior Técnico, Universidade de Lisboa, Av. Rovisco Pais, 1049-001 Lisboa, Portugal; anamoita@tecnico.ulisboa.pt; 4 CINAMIL, Centro de Investigação Desenvolvimento e Inovação da Academia Militar, Academia Militar, Instituto Universitário Militar, Rua Gomes Freire, 1169-203 Lisboa, Portugal; 5 CEFT, Transport Phenomena Research Center, Porto University Engineering Faculty (FEUP), Rua Dr. Roberto Frias, 4200-465 Porto, Portugal; 6 AliCE—Associate Laboratory in Chemical Engineering, Faculty of Engineering, University of Porto, Rua Dr. Roberto Frias, 4200-465 Porto, Portugal

**Keywords:** nanofluids, thermal conductivity, nanoparticles, thermophysical properties, equipment for measuring the conductivity

## Abstract

Fluids containing colloidal suspensions of nanometer-sized particles (nanofluids) have been extensively investigated in recent decades with promising results. Driven by the increase in the thermal conductivity of these new thermofluids, this topic has been growing in order to improve the thermal capacity of a series of applications in the thermal area. However, when it comes to measure nanofluids (NFs) thermal conductivity, experimental results need to be carefully analyzed. Hence, in this review work, the main traditional and new techniques used to measure thermal conductivity of the NFs are presented and analyzed. Moreover, the fundamental parameters that affect the measurements of the NFs’ thermal conductivity, such as, temperature, concentration, preparation of NFs, characteristics and thermophysical properties of nanoparticles, are also discussed. In this review, the experimental methods are compared with the theoretical methods and, also, a comparison between experimental methods are made. Finally, it is expected that this review will provide a guidance to researchers interested in implementing and developing the most appropriate experimental protocol, with the aim of increasing the level of reliability of the equipment used to measure the NFs thermal conductivity.

## 1. Introduction

In recent years, the potential of applying nanofluids (NFs) in different engineering fields [1,2,3,4,5,6,7,8,9,10,11,12,13,14,15,16,17] have been increasing substantially since the use of NFs in devices from the micro- to the macroscale level. NFs are basically colloidal mixtures of nanoparticles (NPs) with a base fluid [10,11,12]. The introduction of NPs in base fluids has been strongly explored in recent decades, for several industrial applications [12]. That addition results not only in a significant increase in the fluid thermal conductivity [13] but also in an improvement of the convective heat exchanges. However, when it comes to measuring the thermal conductivity of these thermofluids, some experiements have shown controversial results. According to Souza et al. [14] and Barbés et al. [15], experimental studies have shown that thermal conductivity of NFs depends on many factors, such as particle material, particle size and shape, particle volume fraction, agglomeration of particles, base fluid material, pH value, temperature, and additives.

**Figure 1:**
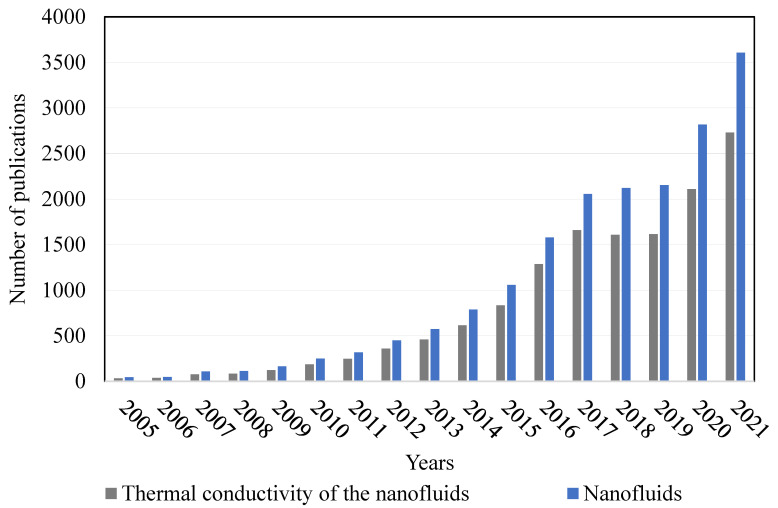
Number of scientific articles presented in the ScienceDirect database from the year 2005 up to 2021.

The increasing interest in the research performed with NFs can be observed in the number of publications presented in the database at Figure 1. The database used was ScienceDirect, where the keywords NFs and thermal conductivity of NFs were used. Figure 1 shows that NFs start to be an interesting topic from the beginning of the 21st century, and that interest has been increasing until the present moment. It can be also seen that the study of the thermal conductivity of NFs is a topic that has been gaining interest by the researchers using NFs.

There are many methods to measure the thermal conductivity of liquids. However, there are three kinds that are the most often used, i.e., the transient, steady-state, and thermal comparator techniques. Some of those will be analyzed in this review work. As demonstrated by Paul et al. 2010 [16], fluids do not have a definite shape, size, and cross-sectional area, which makes it difficult to measure thermal conductivity. In the case of NFs, the complexity to have a homogeneity mixture, the strict control of the flow and temperature experimental conditions and the analyses of thermal transport mechanisms in this type of fluid are even more challenging.

This review is organized as follows: Section 2 presents the main experimental techniques used to measure the thermal conductivity of NFs; Section 3 presents how characteristics and thermophysical properties of NPs, temperature and concentration, and NFs preparation process and use of surfactants influence on the measurements; Section 4 shows the thermal conductivity comparison between different experimental methods; Section 5 compares experimental methods with theoretical methods; and, finally, in Section 6 the main conclusions are drawn.

## 2. Techniques for Thermal Conductivity Measurements of NFs

**Figure 2:**
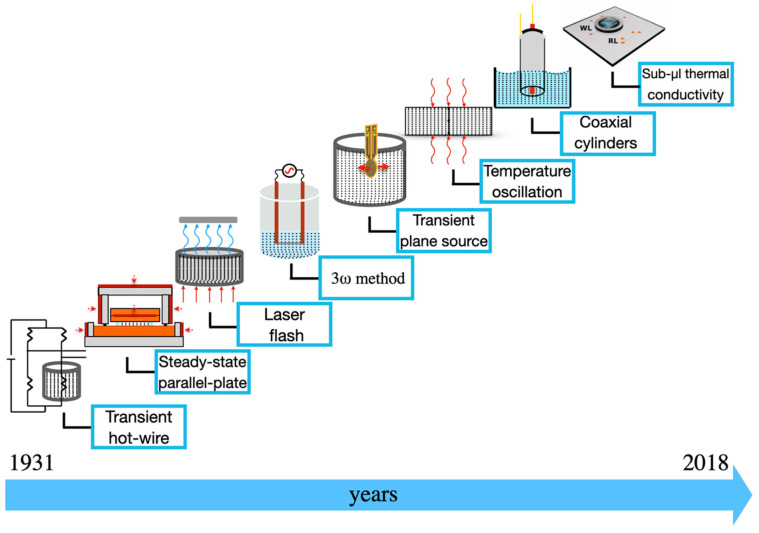
Schematic diagram of the main techniques to measure the thermal conductivity of NFs.

Over the decades, many techniques have emerged to measure the thermal conductivity of liquids. Initially adapted from the devices developed to measure the conductivity of other materials, such as solids, they have been refined to eliminate the convection caused by fluids. More recently, a new transformation of the methods was required due to the growth of research involving NFs. In this section, some of these techniques, as shown in Figure 2, will be presented and discussed.

The techniques shown in Figure 2 are:

- Transient hot-wire that was created Stâlhane and Pyk [17];
- The steady-state parallel-plate method that was developed by Challoner and Powell [18];
- Laser flash method proposed by Parker et al. [19];
- The 3ω method that was presented by Cahill and Pohl [20];
- The transient plane source method that was presented by Gustafsson [21];
- The temperature oscillation that was described by Czarnetzki and Roetzel [22];
- The coaxial cylinders method that the proof of concept was performed by Schiefelbein et al. [23];
- Sub-μL that was recently developed by López-Bueno and co-workers [24].

In the work of Assael et al. [25], the authors have shown that, during the XIX century, a series of experiments with wires heated in gases began to appear in the literature, which have promoted the debate over the heat transfer in gases. At that time, the conduction in gases was questioned and has extended its doubt to liquids as well. The debate was ended, when Assael team [25] showed, with a wide acceptance of the works performed by James Clerk Maxwell [26,27], the calculation of the theoretical value of the thermal conductivity of a gas. Maxwell also has shown the dependence of the thermal conductivity on the temperature and pressure. After that, it is very likely that the first devices to measure the thermal conductivity of liquids have undergone adaptations from the techniques used to measure solids, powders, and some gases.

To perform the calibration of these devices the most frequent liquids, used as a reference, are the toluene and water [28], as the uncertainty for these fluids is smaller than 2% at a 95% confidence level. Other fluids, such as n-heptane [29] and benzene [30], are also common reference fluids but the uncertainty is slightly higher. Additionally, the compounds from the alcohol family are also widely used to calibrate several commercial measurement instruments, such as ethylene glycol, propylene glycol, and glycerol [31]. It is well known that the thermal conductivity of these alcohols, just like water, increases with the temperature, but has the unique advantage of having a higher boiling point. In addition, it has a higher viscosity, so conduction heat transfer dominates over convective heat transfer mechanism [31,32]. So, like in any other experimental technique, measurement and calibration procedures must be adequate to the working conditions and ranges of the system.

### 2.1. Transient Hot-Wire (THW) Technique

Studies with heated wires started in 1780 and measurements of the thermal conductivity of gases date back to 1781. Nevertheless, only in 1888 the first real hot-wire instrument was introduced by August Schleiermacher. In this work, he used a horizontal platinum (Pt) wire, with 0.4 mm in diameter and 32 cm in length, secured by a metallic spring in the center of a glass cylinder, to measure the thermal conductivity of gases. Sophus Weber reported in 1917 some of the errors present in the measurements from the work of Schleiermacher and suggested the vertical positioning of the wire, to reduce convection effects [33].

**Figure 3:**
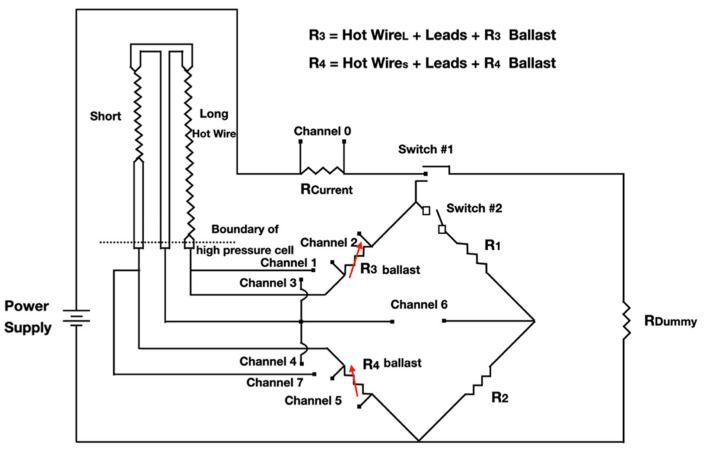
Schematic representation of a TWH installation with a Wheatstone bridge (adapted from Roder [35]).

The first transient hot-wire instrument was proposed by Stâlhane and Pyk in 1931 [17], initially to measure the thermal conductivity of solids, powders, and some liquids. Currently it has been used in NFs. The technique uses a probe that is inserted into the fluid in which it is intended to determine thermal conductivity. The probe has two important functions: supplying heat to the liquid and also measuring its temperature. The metallic wire, from which the probe is made, is traversed by a constant electric current in order to increase its temperature and, consequently, raising the temperature of the fluid to be measured. The thermal conductivity of the fluid is then obtained from the relative change in the resistivity of the wire, which, in turn, is measured using a measurement system consisting of four resistive wires [17,34]. In Figure 3, there is a schematic representation of the experimental installation of the transient hot-wire method (THW) is shown.

That configuration was proposed initially in 1971 by Haarman [35] that used a Wheatstone bridge to measure the difference between the resistance of two identical wires, only differing in length. This not only improved the experiment duration but also completely eliminated the convection effects [33]. The technique analyzes the relationship between time and temperature rise of the probe, subject to a heat flow. After a short interval, the initiation of the step change, according to Stâlhane & Pyk [17], follows the empirical relation for the temperature difference, Δ*T*, given in Equation (1):

(1) $$\Delta T=\frac{Aq}{k}\ln\left(\frac{r_{o}{}^{2}}{t}+B\right)$$

where, *q* is the heat per unit length, $r_{o}$ is the radius of the wire, *t* is the time, and *k* is the thermal conductivity of fluid. Constants *A* and *B* were obtained from fluids of known thermal conductivity.

Further studies solved the Fourier equation to derive the previous expression, turning it into the “ideal solution” (Assael et al. [25], Equation (2)):

(2) $$\Delta T=\frac{q}{4\pi k}\left[\ln\frac{4\alpha t}{r_{o}^{2}}-0.5772\right]$$

where, *α* is the thermal diffusivity of the medium.

Or, for two distinct times in Equation (3):

(3) $$\Delta T_{1}-\Delta T_{2}=\frac{q}{4\pi k}\ln\left(\frac{t_{2}}{t_{1}}\right)$$

The ideal solution considers the hot-wire in the THW apparatus as infinitely long and with infinitely high thermal conductivity and zero heat capacity which transfers the heat radially into an infinite fluid [36,37].

In 1976, Healy et al. [37], the theory behind the THW measurements was presented. The governing equations were solved by approximation to an ‘‘ideal’’ solution so that the thermal conductivity could be determined from the slope of the temperature rise against the time of electrically heated wire [37,38].

The effect of convection could be eliminated by the apparatus design and other corrections could be applied during the experiment, while others were rendered negligible. For instance, the radiative heat transfer at high temperatures could be corrected analytically [33].

**Figure 4:**
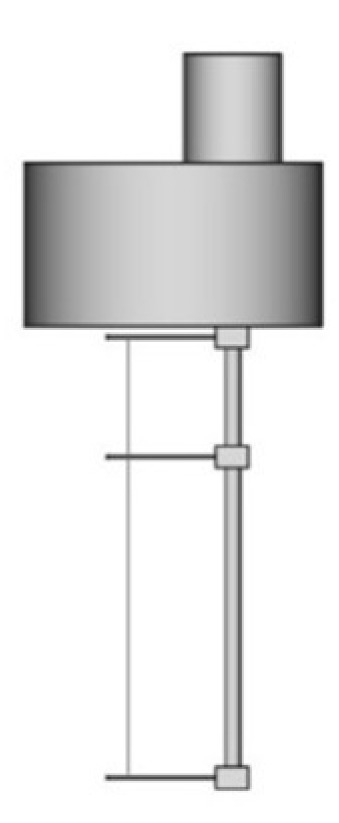
THW probe of tantalum wire with short and long wire placed on top of each other (adapted from Antoniadis et al. [39]).

Most of the THW apparatus used a platinum wire as the hot-wire. In 1982, tantalum started to be used since it could be anodized in situ and, consequently, form a thin insulator layer of tantalum pentoxide. Figure 4 shows an example of a THW probe with a tantalum hot-wire. The same material also started to be used for wire supports to hold the hot-wire. The analytic solution that described an ideal solution was replaced by finite element models that allowed the full representation of the geometry of the THW device [33,39].

In sum, the conditions for good measurements with THW technique are the use of two wires of different length to cancel end effects, the wires should be insulated and very thin, preferentially less than 30 μm diameter. The measurements should have a duration of 0.1 s to 1 s and the temperature should rise less than 4 K during the measurement [39]. Although, Antoniadis et al. [39] have published several recommendations to perform accurate transient hot-wire measurements, many studies reported in the literature did not follow those specifications, which has led to a series of divergent results on the measurement of the nanofluids thermal conductivity. However, in this review it was also decided to include the research works that did not follow the protocol proposed by Antoniadis et al. [39] as the intention of the present review is to bring out the plurality of results, in order to highlight these problems and contribute to improve the procedures and techniques discussed here. So, several studies have used the THW technique to measure the thermal conductivity of different NFs.

Some authors have built their own apparatus using Pt as the hot-wire [34,39,40,41,42,43,44,45,46,47,48,49]. The wires had a diameter ranging from 50 to 100 µm and a length from 140 to 215 mm and were commonly insulated by a Teflon layer. The NPs tested included iron (Fe), titanium dioxide (TiO_2_), aluminum oxide (Al_2_O_3_), zinc oxide (ZnO), copper oxide (CuO), silicon dioxide (SiO_2_), multiwalled carbon nanotube (MWNT), and fullerene, with base fluids water, ethylene glycol (EG), mixtures of the previous, transformer oil, mineral oil, and decene [34,39,40,41,42,43,44,45,46,47,48,49]. The maximum concentrations used were 8 vol.% and 7 wt.% and the temperatures varied from room temperature to 70 °C. All studies reported an increase in thermal conductivity with the increase in temperature and with the particle concentration in the fluid, except for the case of the water-based fullerene nanofluid where thermal conductivity was lower than that of base fluid, as noted by [42]. 

Liu et al. [36] also verified the increase in the thermal conductivity of NFs by applying an electric field, while ref. [50] investigated the effect of the particle size, reporting a positive effect in the thermal conductivity with the reduction in the size. That effect is more pronounced when the volume fraction is very low.

Other authors used the commercial equipment KD2 Pro thermal properties analyzer that contains a probe with a Pt hot-wire that is fully immersed in the fluid during the measurements [51,52,53,54,55,56]. The NFs had water as a base fluid and the NPs used were silver (Ag), silicon carbide (SiC), graphene oxide (GO), TiO_2_, Al_2_O_3_, Fe–Si hybrid, and CuO. An increase in thermal conductivity with the increase in temperature and with particle concentration in the fluid was also reported by the authors and Kalantari and Hashim [51], who verified that a decrease in particle size leads to an increase in the thermal conductivity [51,52,53,54,55,56]. Gangadevi et al. [56] used different sonication times for the preparation of the NFs and verified an increase in thermal conductivity with the increase in sonication time. Mahbubul et al. [54] repeated the measurements for 0.5 vol.% of Al_2_O_3_–water NFs after 30 days of storage and no significant changes in thermal conductivity were registered.

Other commercial equipment was used by Aparna et al. [57], that measured the thermal conductivity of aqueous α-Al_2_O_3_, γ-Al_2_O_3_, and Ag NFs using LAMBDA apparatus (Flucon fluid control GmbH). The equipment uses a 100 μm diameter and 45 mm long Pt wire on the probe. The thermal conductivity was reported to improve with the increase in volume concentration for all the NFs [57]. The laser flash method was also used but the results were significantly lower than the ones obtained by THW [57].

Other authors used different materials for the hot-wire of the THW apparatus. Azarfar et al. [58] constructed a device with a copper wire of 2 mm diameter with a 0.01 mm polyester coating thickness as the hot wire. Additionally, direct and alternating electrical currents were used to heat the wire and unbalance the Wheatstone bridge, respectively. The device was tested with deionized water and EG at different temperatures and the uncertainty of the measurements was around 1%.

Minakov et al. [59] investigated the thermal conductivity of a nanofluid with alumina NPs suspended in ethylene glycol and the hot wire was a copper wire of 80 mm length and 75 μm diameter and the duration of each measurement was 10 s. 

Ebrahimi and Saghravani [60] investigated the effect of a magnetic field on the thermal conductivity of water based Fe_3_O_4_/CuO hybrid nanofluid using THW technique. A metallic Ag wire was used as hot wire and magnetic fields of 0.1 and 0.2 T were applied to the samples during the measurements. The results showed that an increase in the particle concentration and magnetic field improved the thermal conductivity of the nanofluid [60].

There are some variations of the THW technique, namely the liquid metal transient hot-wire technique and the transient short hot-wire technique. The first uses a mercury-filled glass capillary as the hot-wire and is used for measurements with electrically conducting liquids [61]. The second technique has a shorter hot-wire than in the regular THW and is indicated for measurements with highly corrosive liquids [16]. The transient short hot-wire technique measures, simultaneously, the thermal conductivity and thermal diffusivity of liquids, gases, or powders. 

As in the THW technique, a metal wire serves as heating unit and as an electrical resistance thermometer. The values of thermal conductivity and diffusivity are obtained by correlating the experimental data with numerically simulated values based on a two-dimensional heat conduction model. The method of data extraction depends if the correlation between temperature rise and logarithmic heating time is linear or non-linear [62].

The THW technique is faster than other techniques, has a simple design, and natural convection errors can be eliminated experimentally [16]. Nevertheless, ionic liquids, NFs, and molten salts still present a challenge to the thermal conductivity measurements. Many studies used simplified versions of the fundamental THW technique, which could explain some variations in the results [31,37]. However, Peralta-Martinez [63], presented a new instrument for measuring the thermal conductivity of molten metals and salts based on the transient hot wire technique where they have obtained satisfactory results with an estimated uncertainty of ±2%. This device was able to overcome both convection and thermal radiation problems. The mentioned problems are recurrent in the traditional instrument.

Some of the problems arise from the electrical conducting properties of the fluids. The contact between the fluid and the wire of the probe/hot-wire can generate secondary path flows of current; the fluid can polarize or deposit at the surface of the wire and the dual path conduction can affect the automatic Wheatstone bridge; regardless, the thermal conductance of hot-wire/nanofluid interface should be measured [64].

### 2.2. Steady-State Parallel-Plate Method

**Figure 5:**
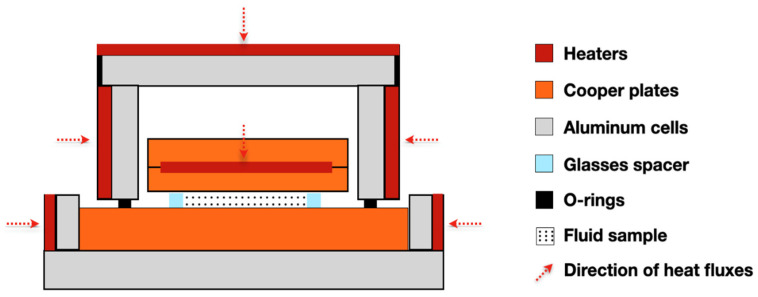
Illustrative scheme of the steady-state parallel-plate method.

Created by Challoner and Powell [18], the parallel-plate method in steady-state was developed to determine the thermal conductivity of liquids. The heating plate is composed of two copper discs and the internal faces of the discs receive a spiral coil of heating wire evenly distributed and have two thermocouples embedded near the outer surface of each disc and glass spacer [65]. Thus, the total heat supplied by the main heater flows through the liquid between the upper and lower copper plates. The general thermal conductivity between the two copper plates, including the effect of the glass spacer, can be calculated from the equation of one-dimensional heat conduction relating the power of the main heater. A scheme of the apparatus can be seen in Figure 5.

To avoid heat loss from the fluid to the surrounding, guard heaters are used to maintain a constant temperature of the fluid. So, the conductivity of the liquid ($k_{l}$) is given by Equation (4) [18]:

(4) $$k_{l}=\left(\frac{0.964\;I_{o}V_{o}d}{t}-k_{s}A_{s}\right)/A_{l}$$

where, $I_{o}$ and $V_{o}$ are the current and potential, respectively, supplied by a potentiometer to the hot-plate heater; $k_{s}$ is the thermal conductivity of the material constituting the pieces; $A_{s}$ is the total cross-sectional area of the distance pieces; *t* is the observed temperature difference between the hot and cold plates; *d* is the thickness of liquid; and $A_{l}$ is effective area of the liquid. This is valid if $A_{l}$ equals $A-A_{s}$, where *A* is obtained by application of the Schwarz–Christoffel theorem.

Wang et al. [66] measured by a steady-state parallel-plate method the effective thermal conductivity of mixtures of fluids and nanometer-size particles. The tested fluids were two types of NPs, Al_2_O_3_, and CuO, dispersed in water, vacuum pump fluid, engine oil, and EG. The researchers showed that the experimental results for the thermal conductivities of mixtures are higher than those of base fluids.

Shalkevich et al. [67] synthesized spherical gold NPs with different sizes (from 2 to 45 nm) and prepared stable gold colloids in the range of volume fraction from 0.00025 to 1.0. The authors used different methods to measure the thermal conductivity. One of them was the steady-state parallel-plate method, and despite an innumerous range of parameters tested (e.g., NPs size, concentrations, temperatures), there was not a significant anomalous enhancement of thermal conductivity. 

### 2.3. Laser Flash Method (LFM)

**Figure 6:**
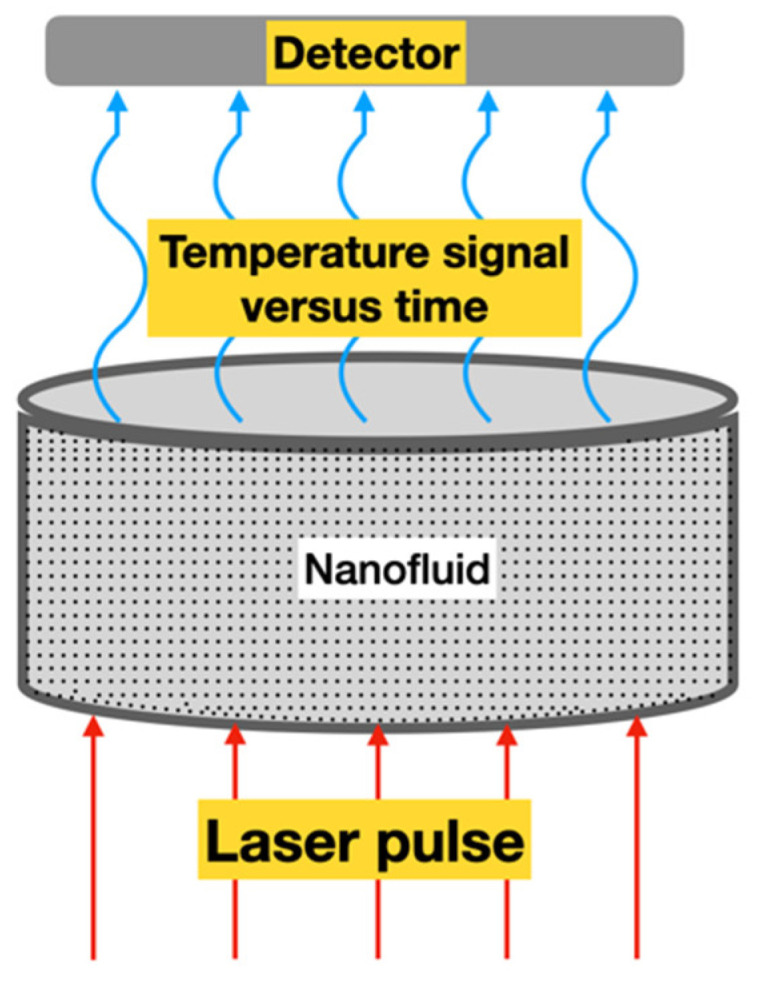
Laser flash measurement principle: an energy/laser pulse (red) heats the sample (gray) containing the nanofluid on the bottom side and a detector detects the temperature signal versus time on the top side (blue).

The laser flash method (LFM) is considered an advanced technique of thermal conductivity measurement and it uses a laser beam as a heat source. It was initially proposed by [21] to measure the thermal diffusivity of solids and, consequently, the thermal conductivity. During the test, the front face of a small sample of the material receives a pulse of heat from radiant energy that increases the temperature on the opposite side of the sample (rear) while recording its value, see Figure 6.

This is an improvement over the transient hot-wire method because it essentially eliminates the effects of convective heat transport during measurement [68]. The analytical solution for the LFM considering one-dimensional thermal conduction is given by Equation (5) [61]:

(5) $$\Delta T=\Delta T_{m\;}\left[1+2\;\sum_{n=1}^{\infty }{\left(-1\right)}^{n}\;exp^{\left(\frac{-n^{2}\pi^{2}\alpha t}{L^{2}}\right)}\right]$$

Here, *L* is the thickness of the sample, ΔT is the temperature rise, $\Delta T_{m\;}$ is the maximum temperature, *t* is the time after pulse heating, and α is the thermal diffusivity [69].

To estimate the thermal diffusivity of the sample, Parker et al. [22] derived a formula from the half-rise time, corresponding to the time necessary for the temperature rise on the opposite side of the surface to reach half of its maximum value (Equation (6)):

(6) $$\alpha =0.1388L^{2}/t_{1/2}$$

where, $t_{1/2}$ is the time required for the back surface to reach half of the maximum temperature rise, i.e., $\Delta T/\Delta T_{m\;}=1/2$. So, the thermal conductivity is found from the relationship presented in Equation (7):

(7) $$k=\alpha \rho c_{p}$$

where, ρ is the mass density and $c_{p}$ is the specific heat.

In 2012, Yang et al. [70], despite using nanodiamond particles with high thermal conductivity, anomalous increases in thermal conductivity measured by the LFM were not observed. The thermal conductivity of nanofluid increases with the increasing particle concentration, such as those predicted by Maxwell [71] and Bruggeman [72] models.

Zeng et al. [73] used the LFM to measure the thermal conductivity of oil-based MoS_2_ NFs. These authors evaluated the heat transfer oil-based NFs, with the mass fraction of lipophilic NPs varying from 0.25 up to 1.0, ranging the temperatures from 40 to 200 °C. They found that these NFs have higher thermal conductivity and the thermal conductivity enhancement increased, not only with an increasing mass fraction of NPs, but also with increasing temperature in the range from 40 to 180 °C. In addition to that, the experimental results show a 38.7% enhancement of the thermal conductivity of MoS_2_ nanofluid with only 1.0% mass fraction at 180 °C.

More recently, Park et al. [74] measured the thermal conductivity of Li_2_TiO_3_ pebble bed by LFM. The results obtained by them showed that the thermal conductivity of the Li_2_TiO_3_ pebble bed with packing factor of 57% increased with increasing temperature up to 500 °C and was saturated to 1.3 W/m/K at more than 500 °C.

### 2.4. 3ω Method

**Figure 7:**
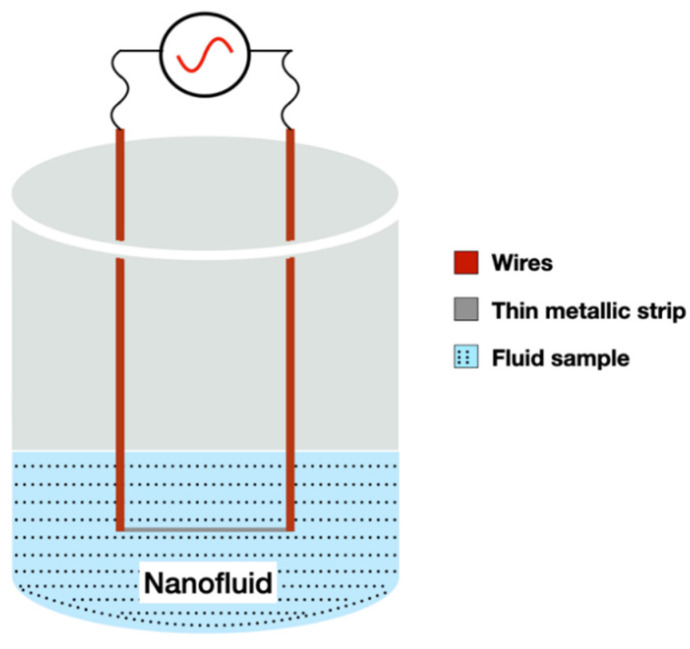
Schematic representation of the 3ω method.

The 3*ω* method uses a thin metallic strip that work as a heater and thermometer, similar to the transient wire method. The strip is deposited on the sample surface to measure thermal conductivity in sequence, see Figure 7.

For the performance of the test, an alternating current, with an angular modulation frequency *ω*, is applied to the metal strip causing Joule heating with power *P* at frequency 2*ω*, as it can be seen in Equation (8) [75]:

(8) $$P=R\cdot I^{2}=R\cdot I_{o}{}^{2}\cdot cos^{2}\left(\omega t\right)=R\cdot I_{o}{}^{2}\frac{1}{2}\cdot \left(1+\cos\left(2\omega t\right)\right)$$

where, *R* is the resistance, *I* is the current, and $I_{o}$ is the peak current. According to Bogner et al. [76], this results in a voltage oscillation along the heating resistor with a third harmonic which depends on the temperature oscillation of the heater.

Cahill et al. [77] showed the exact solution from an infinitely narrow line-source of heat, on the surface of an infinite half-volume at a distance $r={\left(x^{2}+y^{2}\right)}^{1/2}$, as in Equation (9):

(9) $$\Delta T\left(r\right)=\frac{P}{l\pi k}K_{o}\left(qr\right)$$

*P*/l is the amplitude of the power per unit length, generated by the frequency 2*ω* in the line source of heat; *k* is the thermal conductivity of the half-infinite volume;$\;K_{o}$ is the modified Bessel function of zeroth-order and 1/*q* is the thermal wave penetration depth, as defined in Equation (10):

(10) $$\frac{1}{q}=\sqrt{\frac{D}{2i\omega }}$$

where, $D=k/\rho c_{p}$ is the thermal diffusivity, ρ is the mass density, and $c_{p}$ is the specific heat. In respect to the finite width of a deposited strip on a substrate, ref. [77] showed that the temperature amplitude averaged over the heater width can be represented by Equation (11):

(11) $$\Delta T=\frac{P}{l\pi k}\int_{0}^{\infty }\frac{sin^{2}kb}{{\left(kb\right)}^{2}{\left(k^{2}+q^{2}\right)}^{1/2}}dk$$

where, *b* is the half heater width.

Turgut et al. [78] used the 3*ω* method to measure the thermal conductivity and effective viscosity of TiO_2_ water-based nanofluid for temperatures between 13 and 55 °C. They conclude that there is no influence of temperature in the relative thermal conductivity, but, on the other hand, thermal conductivity increases with the increase in volume fraction (0.2 to 3 vol.%).

Karthik et al. [79] used a 3*ω* measurement technique in a suspended micro-wire to analyze the thermal conductivity of CuO/DI-water NFs for 0.025, 0.05, and 0.1 of nanoparticle volume fraction at temperatures of 15, 25, and 35 °C. They obtained an overall enhancement of thermal conductivity over the DI-water for the tested conditions from 13% to 25%.

### 2.5. Transient Plane Source (TPS) Method

**Figure 8:**
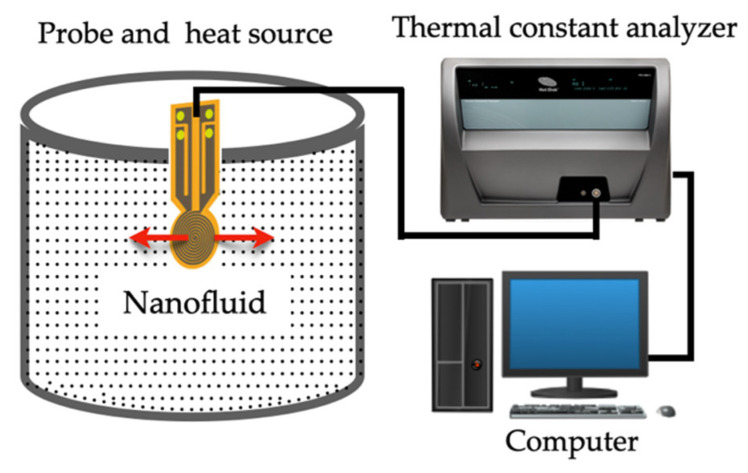
Illustration of the transient plane source (TPS) technique: red arrows represent the direction of the heat flux (adapted from Lin et al. [80]).

The transient plane source (TPS) technique has the advantage to simultaneously determine the thermal conductivity, thermal diffusivity, and specific heat capacity from a single measurement. Once again, similarly to other techniques, the hot disk sensor itself, serves as both a heat source and a temperature sensor. The method employs a sensor of electrically conducting nickel, reinforced by layers of insulating Kapton. To carry out the measures, the sensor is placed between two identical samples and a current is applied to the sensor, which generates heat at the same time which the sensor monitors the temperature. Figure 8 represent the scheme of the TPS technique, adapted from Lin et al. [80].

Between the temperature responses versus the time, it is possible to determine the thermal conductivity of the material by Equations (12) and (13) using the inverse of thermal conductivity 1/*k* [81].

(12) $$\Delta T\left(\emptyset \right)=\frac{Q}{\pi^{1.5}rk}\;D\left(\emptyset \right)$$

(13) $$\emptyset =\sqrt{\frac{t\alpha }{r^{2}}}$$

Here, *r* is the sensor radius, D(∅) is as dimensionless theorical expression of the time dependent increase describes heat conduction of the sensor.

However, as Ma et al. [82] referred, in NFs case (i.e., liquids) measurement convection is the biggest problem. Therefore, in the several studies presented in the literature that use TPS techniques, the authors do not account for the effects of natural convection during the measurement.

Cabaleiro et al. [83] measured experimentally the thermal conductivity and dynamic viscosity of ZnO/(EG + W) NFs with 1.0, 2.5, and 5 nanoparticle mass concentrations. They showed that the thermal conductivity enhancements for the 5 wt.% reach values of 8.3% and also, dynamic viscosity rises strongly with nanoparticle concentration.

In the study performed by [82], the authors sought to determine the thermal conductivity of propylene glycol with different concentrations of silicon dioxide (SiO_2_) NPs and also evaluated dispersion stability of NFs after 10 thermal cycles. The concentration of SiO_2_ was measured at 20 °C before and after thermal cycling and the main results obtained were: (i) 0.5% mass concentration of SiO_2_ exhibited no enhancement in thermal conductivity compared to pure propylene glycol; (ii) 0.75, 1, and 1.5% mass concentration of SiO_2_ the fluid exhibited an average thermal conductivity enhancement of 15%; and (iii) the enhancement remained constant with thermal cycling. In other words, the effects of sensor power and the measurement period has a negligible effect on the measurement [82].

### 2.6. Temperature Oscillation Technique

The temperature oscillation technique to measure the thermal diffusivity (and, consequently, the thermal conductivity) of a fluid, according to Bhattacharya et al. [84]*,* consists of filling a cylindrical volume with the fluid, applying an oscillating temperature boundary condition at the two ends of the cylinder, and measuring the amplitude and phase of the temperature oscillation at any point inside the cylinder. Therefore, from the amplitude and phase values of the temperature oscillations at the ends, and at the point inside the cylinder, it is possible to calculate the fluid thermal diffusivity. However, Bhattacharya et al. [85] had previously called attention to the most important parameters involved in this technique that are the time period and the amplitude of the temperature oscillation: (i) if the time period of the oscillation is small, the oscillation would die out in the middle and no useful measurement can be performed; (ii) on the other hand, if the time period is too large, there would be no phase difference between the thermocouples that measure the temperature; additionally, (iii) if the amplitude is too high, an onset of natural convection might take place and, consequently, the result will be erroneous.

The measurement principle behind the temperature oscillation technique was describe by Czarnetzki and Roetzel [22] solving the energy Equation (14) to obtain the thermal diffusivity and the thermal conductivity:

(14) $$\frac{\partial T}{\partial t}=\alpha \;\nabla^{2}T$$

where, *T* is temperature, *t* is time, and α is the thermal diffusivity. The solution of Equation (14) depends upon specimen geometry and boundary conditions. At the nonadiabatic surfaces of the specimen, periodic temperature oscillations are generated with the period $t_{p}$ and the constant angular frequency, ω, is calculated by Equation (15):

(15) $$\omega =\frac{2\pi }{t_{p}}$$

**Figure 9:**
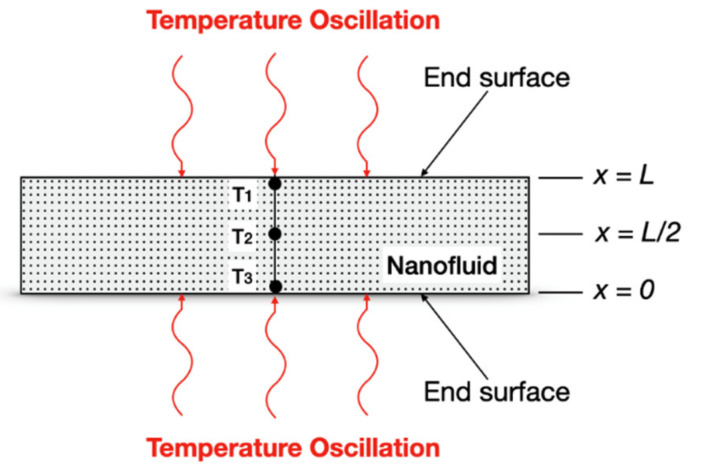
Diagram of the thermal conductivity measurement system with temperature oscillation technique (adapted from Bhattacharya et al. (2004) [82]).

Considering a cylindrical fluid volume for analysis, such as used by Bhattacharya et al. [85] (shown in Figure 9), to measurement the thermal conductivity of NFs it is possible to define the non-dimensional space and time coordinates as the following:

(16) $$\xi =x\;\sqrt{\frac{\omega }{\alpha }}$$

Introduction of the dimensionless time:

(17) τ=ω t

Using Equations (16) and (17), Equation (14) can be rewritten as Equation (18):

(18) $$\frac{\partial^{2}T}{\partial \xi^{2}}=\frac{\partial T}{\partial \tau }$$

In general cases, there is an input of same frequency but with different amplitude and phase at x = 0 and at x = *L*, the boundary conditions are given by Equations (19) and (20):

(19) $$T\;\left(\;\xi =0,\;\tau \;\right)=T_{m}+u_{o}\;\cos\left(\tau +G_{o}\right)$$

(20) $$T\;\left(\;\xi =L\;\sqrt{\frac{\omega }{\alpha }},\;\tau \;\right)=T_{m}+u_{L}\;\cos\left(\tau +G_{L}\right)$$

Here, $T_{m}$ is the mean temperature, $u_{o}$ is the amplitude of oscillation at x = 0, $u_{L}$ is the amplitude of oscillation at x = *L*, $G_{o}$ is the phase of oscillation at x = 0, and $G_{L}$ is the phase of oscillation at x = *L.* For steady periodic conditions, Equation (18) can be solved using the boundary conditions given by Equations (19) and (20) with the method of Laplace transforms where the solution in complex form is presented by [86] through Equation (21):

(21) $$T\;\left(\;\xi ,\;\tau \;\right)=T_{m}+\frac{u_{L}e^{iG_{L}}\;\sin h\;\left(\xi \sqrt{i}\right)-u_{o\;}e^{iG_{o}}\sinh[\left(\xi -\xi_{L}\right)\sqrt{i}\;]\;}{\sin h\;\left(\xi \sqrt{i}\right)}$$

When $u_{L}=u_{o}$ and $G_{L}=G_{o}$ means that input oscillations from both the ends have the same amplitude and phase, then it is possible to write a ratio complex amplitude at any point along the length that at any of the ends, B*, is given by Equation (22):

(22) $$B^{*}\left(x\right)=\frac{\sin h\;\left(\xi_{L}\sqrt{i}\right)}{\sin h\;\left(\xi_{L}\sqrt{i}\right)-\sin h\;\left[\left(\xi -\xi_{L}\right)\sqrt{i}\right]}$$

The real phase difference, ΔG, and the real amplitude ratio, $r_{u}$, are expressed in Equations (23) and (24) as:

(23) $$\Delta G=\left(G_{L}-G_{L/2}\right)=\arctan\left[\frac{Im\left(B^{*}\right)}{Re\left(B^{*}\right)}\right]$$

(24) $$r_{u}=\frac{u_{L}}{U_{L/2}}=\sqrt{{\left[Re\left(B^{*}\right)\right]}^{2}+{\left[Im\left(B^{*}\right)\right]}^{2}}$$

Measuring ∆*G* and $r_{u}$, the thermal diffusivity of the nanofluid can be obtained by solving either Equation (23) or (24). Knowing α, the effective density and heat capacity of the nanofluid, it is possible calculate the effective thermal conductivity of the nanofluid [85].

Das et al. [87] using the temperature oscillation technique showed the enhancement of thermal conductivity with particle concentration and temperature. These researchers investigated the water–Al_2_O_3_ NFs for concentrations of 1% and 4% and temperatures of 20, 40, and 60 °C and they have compared with the pure water.

Additionally, in 2003, Das et al. [88] used the same technique for the measurement of thermal diffusivity and, consequently, thermal conductivity of water base fluid NPs of Al_2_O_3_ and CuO as a suspension material. The main results obtained were: (a) for the Al_2_O_3_–water NFs, the effect of temperature on thermal conductivity enhancement was dramatic, as it climbed from 6.5% to 29% (for 1.0 volume particles concentration) and from 14% to 36% (for 4.0 volume particles concentration); (b) for the case of CuO–water NFs, the change rate of enhancement with temperature did not change as much with concentration as the observed for Al_2_O_3_–water nanofluid; and, last, (c) for 1% and 4% volume particle concentrations there was a considerable increase in the enhancement from 21 to 51 °C (for 1 vol.% at 21 °C the enhancement in only about 2%, but at 51 °C this value increased to about 10.8%).

### 2.7. Coaxial Cylinders Method

**Figure 10:**
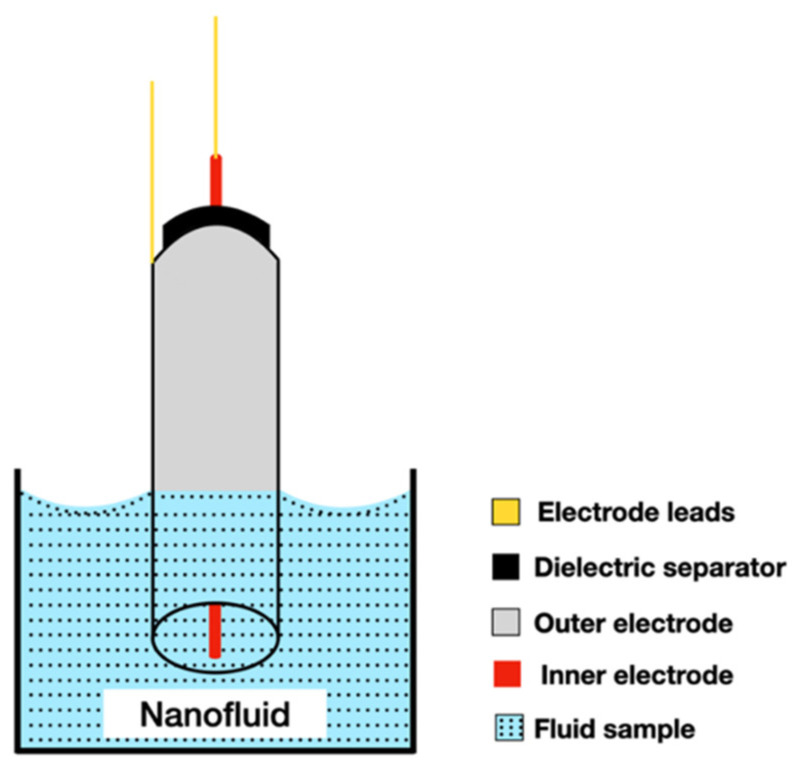
Illustration of the coaxial cylinder method used to measure the thermal conductivity of a liquid (adapted from Schiefelbein et al. (1998) [25]).

The coaxial cylinders technique uses two cylindrical electrodes. The internal electrode is a rod located coaxially inside the electrode of the external tube. The two electrodes are positioned by dielectric separators that never come into contact with the liquid under investigation. The electrodes are immersed in the liquid at an arbitrary initial depth and the AC (alternating current) impedance is measured over a wide frequency range, as is shown in Figure 10.

This process is repeated for several immersions and the electrical conductivity is calculated from the change in conductance measured with the immersion depth [23]. During the procedure it is important to apply very small temperature gradients, thus reducing natural convection.

The working principle of this type of device was described in detail by Tropea et al. [89] and it considers a thin layer of conductivity fluid, *k*, enclosed between two coaxial cylinders of infinite length. The external radius of the inner cylinder is *r*_1_, and the internal radius of the external cylinder is *r*_2_. So, it is assumed that the heat flux is uniformly generated in the inner cylinder and propagates radially through the test sample to the heat sink for the outer cylinder, in steady-state conditions. In this way, the temperatures of the external surface of the inner cylinder and of the internal surface of the outer cylinder will be, respectively, *T*_1_ and *T*_2_. Then, the thermal conductivity is obtained using Equation (25):

(25) $$\kappa \;\nabla^{2}T=0$$

and the amount of heat transferred (*Q*) by conduction per unit time and per unit length through the fluid layer is given by Equation (26):

(26) Q=2πklog(r2r1)(T1−T2)

Tropea et al. [89] also added that, in practice, the length of the cylinders is not infinite and the heat transfer through their ends must be considered. Then, alternatively, if the end pieces are maintained at the same temperature as the inner surface of the outer cylinder, the thermal conductivity of the fluid is obtained from Equation (27):

(27) $$Q=\frac{k}{C}\left(T_{1}-T_{2}\right)$$

where, *Q* is the total amount of heat generated in the emitter and *C* represents a geometric instrument constant that depends just upon the geometry of the coaxial cylinders.

This method is considered suitable for the study of NFs because the measurement is made with very small temperature gradients and with no practical natural convection, as shown by Barbés et al. [90]. In addition, the method allows good temperature control and a very accurate measurement of the heat flow that passes through the sample, as shown by Barbés et al. [15], who performed measurements using the steady-state coaxial cylinders method, with a C80D microcalorimeter (Setaram, France) equipped with calorimetric vessels. Barbés et al. [90] measured thermal conductivity of the Al_2_O_3_–water-based nanofluid at 25 °C, using this technique, and they observed an increase of the thermal conductivity with the increase of the nanoparticle volume fraction.

Barbés et al. [90], Barbés et al. [91]*,* and Barbés et al. [15] measured thermal conductivities of CuO and Al_2_O_3_ NPs dispersed in water and EG, as a function of the particle volume fraction for the temperatures varied between 298 and 338 K, and they observed a thermal conductivity increase for the NFs with increasing of temperature for both NPs.

### 2.8. Novel Experimental Methods

In this section, recent methods that have emerged in the literature will be considered. Some of them are adaptations of traditional methods and others, are just new techniques. A brief overview of the methods will be described and more details can be obtained in the original works.

#### 2.8.1. Modified Transient Plane Source (MTPS)

**Figure 11:**
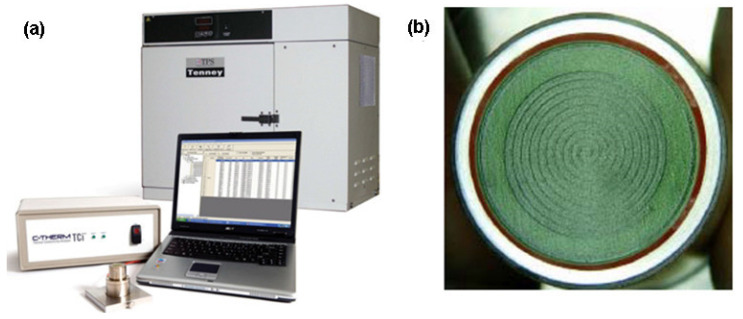
(**a**) TCi thermal conductivity analyzer (foreground), Tenney Jr. Thermal Chamber (background) source and (**b**) the MTPS sensor. Diameter of green surface is 17 mm. (adapted with permission from Harris et al. (2014) [88]).

Harris et al. [92] measured the thermal conductivity of heat transfer fluids using a modified transient plane source (MTPS). The MTPS measure the thermal conductivity using a system composed by: a sensor, control electronics, and computer software (Figure 11a). The sensor has, in its center, a spiral surrounded by a guard ring (Figure 11b), that is responsible for generating heat, in addition to the spiral heater.

The MTPS sensor just measures effusivity in a direct way, so the heat capacity and density of the sample have been known to calculate thermal conductivity. The possibility of having thermal conductivity values without the already mentioned properties, can be solved by using an iterative method m* described in US Patent 6,676,287 [92].

The authors analyzed three distinct fluids, which were distilled water, EG, and transformers oil, and concluded that the MTPS provides an easy way to accurately measure the thermal conductivity and distinguish this form of heat transfer in opposing the impact of convection by: (a) employing the shortest test time in commercially available sensors (0.8 s) and (b) offering a minimal sample volume requirement (1.25 mL), and (c) employing a low-energy power flux to the specimen under test (approximately 2600 W/m^2^) [92].

#### 2.8.2. Extended 3*ω* Method

**Figure 12:**
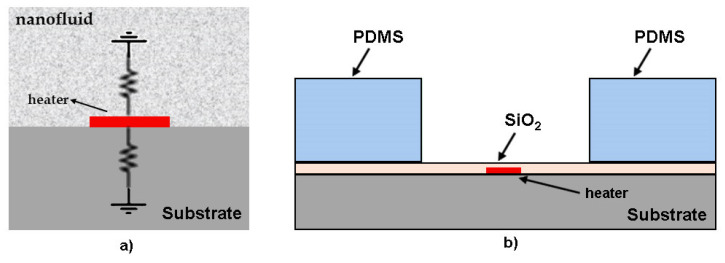
(**a**) Schematic and equivalent thermal circuit of the heater and two semi-infinite mediums of the nanofluid and the substrate; Microfabricated heater device for measuring thermal conductivity of nanofluid and (**b**) cross-section of the heater on 2 mm thick quartz substrate (not to scale) (adapted from Oh et al. [93]).

The first authors in literature who used a type of sub-µL thermal conductivity were Oh et al. [93], but they call their method an extended method from 3*ω* method. In a simplified description of the method, a drop of the nanofluid is placed on a quartz substrate connected to a thin metal heater, as can be seen in Figure 12a.

Since the substrate and the nanofluid layer are modeled as two separate semi-infinite media, their thicknesses must be chosen carefully. The microdevice was microfabricated at the Stanford nanofabrication facility (SNF) by metal deposition and patterning on a 2 mm thick fused quartz wafer (Figure 12b). This micro-sensor is placed inside a temperature-controlled cryostat (Model 330, Lakeshore) and all experiments are conducted at room temperature (21 °C). The metal heater in the microdevice is configured as part of a balanced Wheatstone bridge, and a lock-in amplifier (SR830, Stanford Research) is used to accurately measure the 3*ω* voltage across the metal heater [93]. The temperature oscillation from the measured 3*ω* voltage (*V*_3*ω*_) is calculated by Equation (28):

(28) $$\Delta T_{h\;}=2\frac{V_{3\omega }}{I_{h}}\frac{\partial T}{\partial R}$$

where, *I_h_* is the current at frequency *ω* across the microheater and *∂T*/*∂R* is the ratio of temperature change with respect to the resistance change of the microheater.

Oh et al. [93], calls attention to the fact that the 3*ω* technique can be used to measure thermal conductivity of NFs using a single droplet of volume size. In addition to that, the technique has a significant drawback when measuring NFs with lower thermal conductivity and heat capacity. This occurs because less heat is flowing from the metal strip to the fluid as compared to one solid substrate, for instance, with high thermal conductivity and heat capacity. This causes a reduction in the precision of the device making the 3*ω* signal from the solid substrate more significant than from the fluid.

The first experiments with this method were made by [93], where Al_2_O_3_ NPs were tested with DI-water and EG used as the base fluid. The volume fraction used was 1.0% and 4.0% and it was noticed that thermal conductivity of the NFs increases with the nanoparticle volume fraction. However, there are some discrepancies in their results compared with previous studies from other authors. One of the reasons may be due to sedimentation and aggregation of NPs or NFs preparation.

In 2020, the same author, Oh et al. [94], used this extended method again, with some differences in fabrication of the sensor, to test other NFs. The MWCNTs were suspended in EG in a volume fraction of 0.3%, and the results were in agreement with previously reported values

#### 2.8.3. Sub-µL Thermal Conductivity

**Figure 13:**
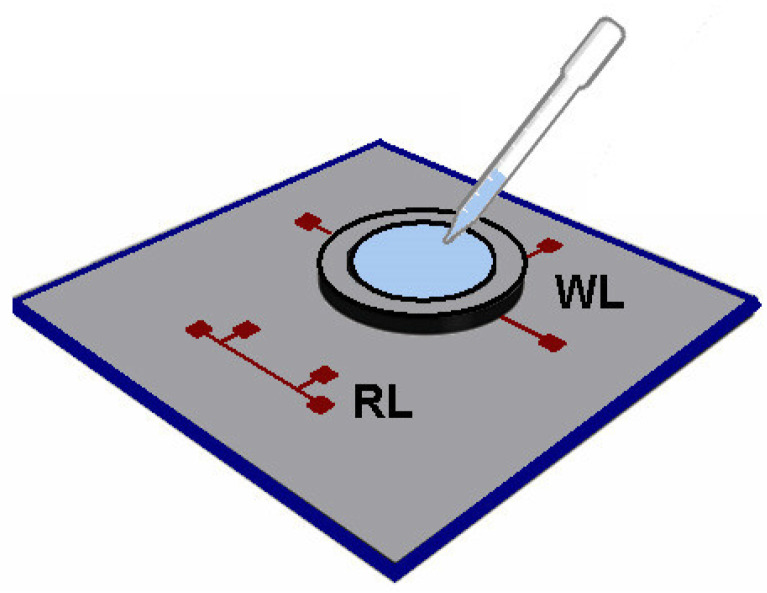
A scheme of the experimental apparatus for the measurement of the thermal conductivity and heat capacity of NFs, where WL and RL represent the working and references line, respectively (adapted from López-Bueno et al. [24]).

The sub-µL thermal conductivity method was developed based in the 3*ω* method, and is capable to simultaneous measurement *k* and *c_p_*. One of its main advantages is the small volume (0.6 µL) of the sample required for the characterization of systems like NFs, in which having a large amount of the dispersed phase is sometimes extremely challenging [24]. The experimental set-up development by [24] can be seen in Figure 13.

To build this sensor it was used a standard photolithography and lift-off to create two identical lines of Cr (10 nm)/Pt (100 nm) deposited on top of a low thermal conductivity glass substrate. The working line (WL) is the one in contact with the liquid, and the reference line (RL) is used as a reference to obtain accurate values of *k* of the glass substrate. A PELCO^®^ silicon disk frame is used to hold the liquid sample, with a thickness of 200 mm and a square aperture of 1× 1 mm, is placed on top of the working line and an AC current was used to provide frequency through the Pt lines [24]. The authors studied thermal conductivities and heat capacities of different fluids at room temperature, and values were satisfactorily in agreement with the results from the literature, with deviations within more or less 10% for most of them.

#### 2.8.4. Steady Flow Method (SFM)

Recently, Xu et al. [95] presented a novel method defined as steady flow method (SFM) that was employed and improved based on the heat transfer of laminar flow theory under the uniform heat flux condition. According to the heat transfer of pipe flow theory, for the uniform constant heat flux boundary condition under the laminar flow, when both flow and thermal boundary layers are fully developed in the pipe, the coefficient of heat transfer is a constant value. In addition to that, the wall temperature and bulk temperature of the fluid increases linearly with the same slope. Based on those conditions, the heat convection of the fluid is mainly a diffusion process in the circle channel, which is only related to the thermal conductivity of fluid and the diameter of pipe and theoretical derivation process can be obtained for the thermal conductivity measurement. So, the thermal conductivity of the working fluid can be solved by Equation (29) [95]:

(29) $$k_{SFM}=\frac{11}{48}\;\frac{Mc_{p}\left(T_{out}-T_{in}\right)}{\pi L\left(T_{w}-T_{f}\right)}$$

where, *M* is mass flow rate (kg/s), $c_{p}$ is specific heat (J/kgK), *L* is length of pipe (m), $\left(T_{w}-T_{f}\right)$ is the temperature difference between wall and fluid and $\left(T_{out}-T_{in}\right)$ is temperature difference between inlet and outlet of test section, on the basis of meeting the assumption that the weight force is neglected. The authors also modified Equation (29) to include the effects of buoyancy and natural convection in the horizontal pipe with laminar flow, presented in Equation (30):

(30) $$k_{ISFM}=\frac{MC_{p}\left(T_{out}-T_{in}\right)}{\pi L\left(T_{w}-T_{f}\right)Nu{\left(k\right)}_{Morcos}}$$

where, $Nu{\left(k\right)}_{Morcos}$ is the *Nu* correlation proposed by Morcos and Bergles [96], then the effective thermal conductivity of fluids are calculated iteratively. So, the method is called iterative steady flow method (ISFM).

Xu et al. [95] measured the thermal conductivity of Al_2_O_3_–water-based NFs under flow condition. The maximum thermal conductivity of NFs with mass concentration of 0.2%, 0.5%, and 1% Al_2_O_3_ showed an increase of approximately 10.5%, 16.7%, and 22.8% compared with the deionized water. Furthermore, the enhancement on thermal conductivity of NFs would be augmented with the fluid temperature. They also showed that the ISFM reduces the influence of natural convection in the processes of measurement, and then leads to improving the measuring accuracy of the thermal conductivity significantly.

A review of the specialized literature showed that the main techniques used to measure the thermal conductivity of NFs have been improved over the years, and most of them still require common protocols for the preparation of NFs, control of conditions of temperature and time interval between tests, in order to minimize the convection effects of the NFs, as well as to guarantee that the measuring equipment operates within a margin of reliability in relation to the material to be analyzed. For this reason, a rigorous theoretical description of the calculation procedures that such equipment provides needs to be known and considered by the operator before the tests. In summary, Table 1 presents the main advantages and disadvantages of the most used methods to measure NFs thermal conductivity.

## 3. Characteristics and Conditions of NFs That Influence Thermal Conductivity Measurements

In this section, it will be presented and discussed how the experimental conditions and changes in the composition of nanofluids influence the measurement of thermal conductivity. The influence of the characteristics and thermophysical properties of NPs are shown in Section 3.1, the temperature and concentration influence are shown in Section 3.2, and Section 3.3 discusses the effect of the NFs preparation process and use of additives.

### 3.1. How Characteristics and Thermophysical Properties of NPs Influence the Thermal Conductivity Measurements

**Figure 14:**
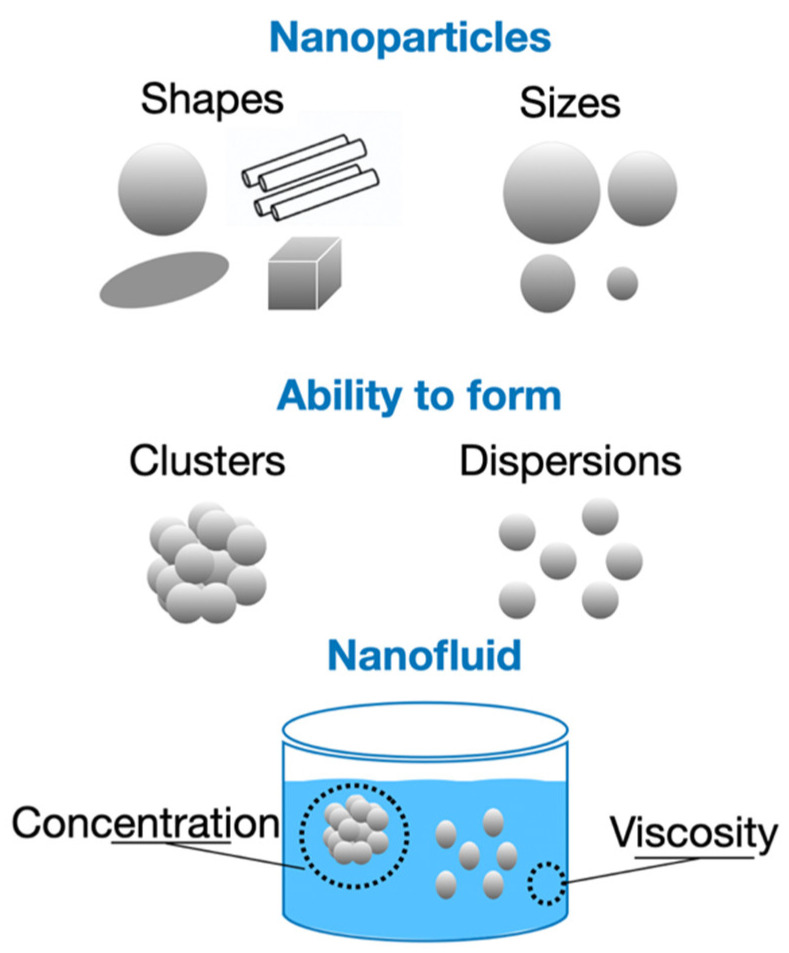
Different features and properties of NPs and base fluid that influence thermal conductivity measurements.

Depending on their thermophysical characteristics and properties, NPs can influence the quality of the thermal conductivity measurements in NFs. In addition to the different shapes that NPs may have, other characteristics, such as density, size, the ability to form clusters or to disperse, the concentration, and also the viscosity of the base fluid, can affect the final measurement of the thermal conductivity of the NFs, as shown in Figure 14.

It is more frequent to find studies involving two types of NPs, spherical and cylindrical, where nanotubes can be considered as cylinders. For example, Xie et al. [99] showed that the NFs formed by the NPs of SiC with cylindrical shape have an increase in thermal conductivity equal to 23%, against 15% compared to spheres for the same volumetric concentration of 4% in EG and in distilled water.

**Figure 15:**
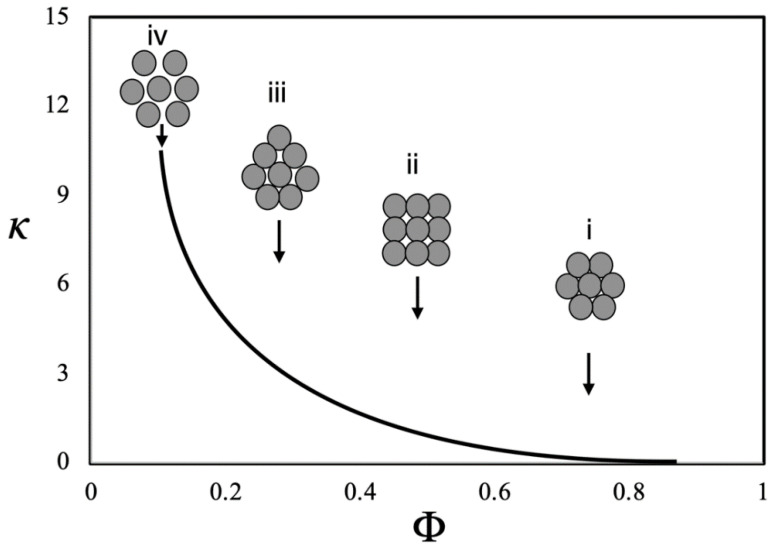
Scheme adapted of diagram proposed by (Eastman et al. [100]) to explain excess thermal conductivity enhancement in NFs. Where κ is the thermal conductivity as a function of the packing fraction of the cluster Φ (ratio of the volume of the solid particles in the cluster to the total effective volume of the cluster).

The clustering of NPs presented by Eastman et al. [100] is one of most important mechanisms for explaining the thermal conductivity improvement in NFs. The authors have described the effect of clustering, indicating possible arrangements of the NPs within the base fluid. In Figure 15, it is a schematic diagram which, according the Eastman team: (i) represents closely packed fcc arrangement of particles; (ii) has a simple cubic arrangement; (iii) a loosely packed, irregular structure of particles in physical contact; and (iv) clusters of particles separated by liquid layers thin enough to allow a rapid heat flow among particles.

In general terms, decreasing the packing fraction, the effective volume of the cluster increases, improving the thermal conductivity. Thus, during a grouping of NPs, a case of total aggregation can be made in which small particles in larger quantities behave similarly to a case with a smaller number of larger particles [101,102].

In the study of [66], it was measured the effective thermal conductivities of fluids (water, vacuum pump fluid, engine oil, and EG) with Al_2_O_3_ and CuO NPs, and it was found that thermal conductivities of nanoparticle-fluid mixtures increase with decreases in the particle size, and that thermal conductivity increase also depends on the dispersion technique.

To evaluate the influence of the nanoparticle size on the thermal conductivity, Vajjha and Das [102] conducted two sets of measurements with ZnO NFs of particle sizes of 29 and 77 nm. Using two different particle volumetric concentrations of ZnO nanofluid in the 60:40 EG/water, the researchers showed that the thermal conductivity ratio is higher for smaller size NPs. For instance, at 305 K the thermal conductivity ratio is 3% higher for 29 nm particle over that of 77 nm particle at a volumetric concentration of 2%. For the 4% volumetric concentration the thermal conductivity ratio is 3.3% higher for 29 nm particle over that of the 77 nm particle. They pointed out that the thermal energy transfer is dependent on surface area and smaller particles of same volumetric concentration provide more surface area for the heat transfer.

### 3.2. How Temperature and Concentration Influence on the Thermal Conductivity Measurements

It is known that the properties of liquids such as specific mass, viscosity, and surface tension, among others, vary with temperature. For this reason, a series of experiments has been developed to show that the thermal conductivity of NFs can also be influenced by the change in temperature of the medium. 

Mintsa et al. [103] measured the effective thermal conductivity of alumina/water and copper oxide/water NFs using a Decagon devices KD2 Thermal analyzer. Measurements were performed for temperatures between 20 °C and 40 °C for various particle volume fractions up to 9%. The results have shown a gradually increase in the effective thermal conductivity with an increase in particle volume fraction and with a decrease in particle size. In addition, for the tested nanofluids, the authors have found a 15% increase in the thermal conductivity for a temperature of 40 °C, when compared with the room temperature (20 °C).

Paul et al. [16] have performed a study on the thermal properties of CuO dispersed in water and EG as a function of the particle volume fraction and at temperatures between 298 and 338 K and the results have shown a growth of the thermal conductivity of NFs with the increase in temperature. The researcher’s team [16] concluded that this behavior is due mainly to the base fluids (water and EG), rather than to the NPs and, as a result, the thermal conductivity enhancement is temperature independent. Barbés et al. [15] have conducted measurements using the steady-state coaxial cylinders method and have obtained experimental results that have shown that the relative thermal conductivity is essentially temperature independent. Another important conclusion of these researchers was that the reported growth of the thermal conductivity of NFs with increasing temperature were due mainly to the base fluids (water and EG) rather to the NPs.

More recently, Riahi et al. [104] have investigated the thermal conductivity of synthesized Al_2_O_3_–water nanofluid prepared by laser ablation in liquid method and they have performed experimental tests for NPs volume fractions of 0.4 vol.% and 0.7 vol.%, at a temperature range between 25 °C and 45 °C. The authors have concluded that the increase in temperature and NPs concentration leads to higher thermal conductivity of NFs, being that the thermal conductivity enhancement was around 8.6% at NPs volume fraction of 0.7 vol.% and temperature of 45 °C. One possible explanation related by them is due to the NPs motion that increases with the temperature and their kinetic energy are more activated.

An increase in the thermal conductivity with the temperature was also noticed by Agarwal et al. [105] and the effect was higher by increasing the concentration. They also showed that, at higher concentrations, thermal conductivity enhancement with temperature has been more salient compared to NFs of lower concentrations. Enhancements of 16.45% and 19.76% on the thermal conductivity was observed for Fe_2_O_3_/water and Fe_2_O_3_/EG NFs of 2 vol.% at 70 °C compared to water and EG base fluids at 10 °C, respectively. At last, according the experimental results of [105], the thermal conductivity enhancement rate was higher for EG base NFs compared to water base NFs, for the same concentration of Fe_2_O_3_ NPs.

### 3.3. How NFs Preparation Process and Surfactants Influence on the Thermal Conductivity Measurements

Buongiorno et al. [106], to explain the theorical inconsistencies of the nanofluid thermal conductivity, cite as possible causes: (i) the broad range of experimental approaches that have been implemented to measure nanofluid thermal conductivity, (ii) the often-incomplete characterization of the nanofluid samples used in those measurements, and (iii) the differences in the synthesis processes used to prepare those samples, even for nominally similar NFs. 

NFs could be manufactured by two different methods, the one-step method and the two-step method. In the first method, the nanostructures are made and dispersed within the base fluid simultaneously. This method avoids the care with the storage of particles, handling, and dispersion, besides minimizing the agglomeration of the NPs and increases the stability of the NFs. However, the high cost and the level of impurities on the nanofluid, that can be considerable and uncontrollable [101].

In most experimental studies, the two-step method is more used. This process is an economic method of production of NFs at large scale [107]. The method uses various kind of materials, such as nanofibers, nanotubes, and nanosheets, among other nanomaterials to produce a dry powder which is the desired nanoparticle. To obtain the dry powder, different techniques can be used, such as mechanical grinding, chemical reaction, condensation of internal gas, or decomposition of organic complexes, as indicated by [107]. However, the principal disadvantage of this method is maintaining the stability of the NFs. For this reason, common solutions to these challenges are ultrasonic vibration (this process is used to speed dissolution by breaking intermolecular interactions of the particles, [107]), changing the pH value of the suspension and addition of surface activators and/or dispersants [108,109].

So, on one hand, NFs can be prepared in the one step method, having more stability, less aggregation, and no need of surfactants, that means better results in the enhancement of the thermal conductivity. On the other hand, the two step method produces NFs with more purity. However, by using this method sonication is always needed to improve the stability. Alternatively, it is possible to add surfactants to the NFs, but this procedure will reduce the enhancement of the thermal conductivity.

In summary, the choice of the type of preparation of nanofluids can compromise the quality of experimental measurements of thermal conductivity. Misconceptions, such as poor characterization of nanoparticle suspensions, poor sample stability, and particle motions and sedimentation during measurements can significantly interfere with the test values and repeatability.

Another doubt among the scientific community is the effect of using surfactants. According to Cakmak et al. [110], they should increase stability and heat transfer, however, recent studies on the effect of surfactants on nanofluid stability and thermal conductivity suggest a quite different situation [110,111]. Using the transient hot-wire technique, Cakmak et al. [110] measured the thermal conductivity of graphene oxide in de-ionized water NFs at 20–40 °C and showed that all NFs without surfactant had higher thermal conductivity than de-ionized water at all temperatures. In addition, an increase in the graphene oxide concentration from 0.01 to 0.2 mass% improved thermal conductivity by 3.05 and 22.03% at 20 °C, respectively. A further increase to 0.25 mass% caused a 23.73% increase in thermal conductivity.

Ouikhalfan et al. [111] realized the thermal conductivity measurements of water NFs with surfactant treated TiO_2_. Two kinds of surfactants, CTAB and SDS, were used. The stability test indicated that water-based nanofluid with modified NPs are found to be stable, with a less than 12% concentration drop over two weeks for CTAB-modified TiO_2_ and 20% for SDS-modified TiO_2_. The thermal conductivity results indicated an enhancement of 10% and 8% for 1.25% of CTAB-treated and SDS-treated TiO_2_, even after 2 weeks from preparation, respectively. The researchers concluded that, despite of presence of the evident nanoclusters, surfactant modified NPs exhibited long lifespan in water-based NFs compared to the unmodified NPs. 

Even more recently, Mustafizur et al. [112] showed some experimental results obtained for the thermal conductivity of NFs with or without the addition of surfactants. In general, the results seem to respect a trend in relation to the use of stabilizers, reducing the thermal conductivity of NFs, which may depend on the amount of surfactant added to the mixtures. The results obtained by them agree with [110] but diverge from [111].

At last, the treatment of the water-based NFs with surfactant can be considered as a promising way to enhance the stability from the colloidal mixtures, however, in relation to thermal conductivity, more studies are needed. One possibility would be to measure the thermal conductivity of the surfactant added only to the base fluid, without the use of nanoparticles, using different concentrations and types of stabilizers in this process. By assessing separately the effects of the surfactant on the base fluid could lead the researchers to formulate a more assertive conclusion about its role on thermal conductivity.

## 4. Thermal Conductivity Comparison between Experimental Methods

The large diversity of techniques to prepare NFs, the different shapes and dimensions of NPs might be one of the main reasons for the difficulty to compare the experimental values measured by thermal conductivity techniques. An effective way of assessing the reliability of the methods is by performing, in the same study, measurements with more than one technique. In this section, experimental studies that compare different techniques to measure the thermal conductivity of their NFs will be presented.

**Figure 16:**
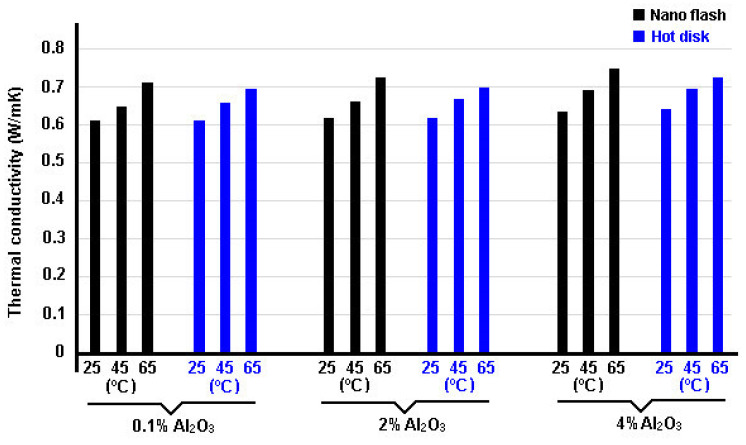
Thermal conductivity data obtained by Buonomo et al. [113] for the Al_2_O_3_-water using the flash and hot disk techniques.

Buonomo et al. [113] measured the thermal conductivities of nanofluid mixtures (alumina/water) using two different methods: the flash and hot disk technique. Thermal conductivity measurements were performed on a nanofluid at 0.1, 0.5, 2, 3, and 4% of volume in a temperature range between 25 °C and 65 °C. In Figure 16, thermal conductivity data obtained by researchers are reported, for both the laser flash and the hot disk methods.

Deviations between the two methods are below or similar to the experimental uncertainty, being the maximum deviation of 5.4% at the higher volume fraction, which showed that there are no difference between one method and another. An enhancement of 13.3% and 10.2% for laser flash and hot disk method, respectively, was found for the nanofluid at 4 vol.%. Lastly, they also showed that the thermal conductivity increases with NPs concentration and with temperature.

Using the same experimental methods adopted by [113] to measure the thermal conductivity of NFs, Zagabathuni et al. [114] showed that when the conductivity of the same nanofluid is measured by the laser method, the enhancement reported is about one order of magnitude lower than when measured by the transient hot-wire method. The authors explained that a small volume (about 50 μL), normally used in the laser flash method, severely restricts the Brownian motion of particles compared to the much larger volume (more than 50 mL) available in the transient hot-wire method. Then, this significant difference in the constraints on the Brownian motion of NPs in NFs affects the frequency of collision with the heat source. 

The differences in the results obtained by [114] had been explained by Ghosh et al. [115] through a collision model that was later improved by Karthik et al. [116]. According to them, during the collisions of NPs with the heat source, rapid heat exchange occurs increasing the temperature of the particles. If the collision frequency per unit area of the heat source and the average thermal energy pickup by the nanoparticle are higher, the increase in the thermal conductivity of the NFs will be more striking.

Aparna et al. [57] made a comparative study of the transient hot-wire and laser flash techniques. However, the authors did not consider identical NFs, but they evaluated the two methods with identical distribution of Al_2_O_3_ and Ag NPs in water. The main results obtained by them were: (i) for the same identical volume fraction, size distribution and shape distribution of NPs, the thermal conductivity enhancement measured by transient hot-wire technique was significantly higher than those measured by laser flash method. (ii) The collision flux of the nanoparticle increased with the increase in perpendicular distance among the NPs and the heat source wall. Such an increase results in higher thermal conductivity in case of transient hot-wire compared to laser flash method, in other words, it was found that the flux of collision of NPs was one to two orders of magnitude lower in the case of laser flash technique as compared to transient hot-wire method. The average collision velocity of the NPs with the heat source depends on the elastic modulus and density of the NPs.

Kostic and Walleck [117] did comparative measurements of the steady-state parallel-plate and transient hot-wire techniques to evaluate the NFs containing silica and alumina NPs. The polymer concentrations chosen by them were 0.02% and 0.05% PVP by weight and 0.02% and 0.05% polyacrylamide by weight (i.e., 100 and 500 wppm), for the silica POLY-NFs; and 0.02% and 0.05% PVP by weight and 0.01% and 0.02% polyacrylamide by weight, for the alumina POLY-NFs. The results obtained for the average thermal conductivity were: (a) the enhancement over the base fluid exhibited by the *silica* POLY-NFs was 1.3% when measured using the steady-state parallel-plate, (b) when measured using the transient hot-wire technique the increases were of 4.4%, and (c) when the thermal conductivity was compared with the base fluid exhibited a enhancement of 3.8% using alumina POLY-NFs measured by the steady-state parallel-plate and 11.4% when measured using the hot-wire technique. 

In 2016, Tertsinidou et al. [118] investigated the thermal conductivities and viscosities of a selection of NFs. The NFs studied were: (a) EG with added CuO, TiO_2_, or Al_2_O_3_ NPs; (b) water with TiO_2_ or Al_2_O_3_ NPs or multiwall carbon nanotubes (MWCNTs); and (c) the use of dispersant also was analyzed. All of the measurements were conducted at 298.15 K. The authors used two techniques to measure the apparent thermal conductivity of NFs, transient hot-wire and using hot disk thermal constants analyzer, which is based on the transient plane source (TPS). In general, both methods used to measure the thermal conductivity of NFs, for all cases studied, there was no significant difference in values between one technique and another. In addition, in this study they have shown that the thermal conductivity increases with the nanoparticle volume.

## 5. Theorical vs. Experimental Models

Some researchers had been comparing results of thermal conductivity from experimental tests with the values predicted by the theoretical models. Lee et al. [50] compared the predictions of thermal conductivity obtained by the Hamilton and Crosser model [119] with experimental results. The NFs used had a base fluid of water or EG and contained aluminum oxide (Al_2_O_3_) or copper oxide (CuO) NPs in a maximum volume concentration of 5%. The measurements were performed at the temperature range of approximately 17–37 °C. The theoretical model was able to predict the thermal conductivity of the Al_2_O_3_ NFs but was inadequate for the CuO NFs. The authors suggested that the difference in results was due to the influence of both size and shape of the NPs on the thermal conductivity.

In 2005, Murshed et al. [40] analyzed experimentally and theoretically the thermal conductivity of water-based NFs with rod-shaped and spherical-shaped of titanium oxide (TiO_2_) NPs. The particles presented the average dimensions of ∅10 nm × 40 nm and ∅15 nm and the concentration of the solutions ranged from 0.5% to 5% in volume. In the experimental tests, the maximum enhancement of the thermal conductivity was nearly 33% for the rod-shape particles and close to 30% for the spherical-shaped particles, over the base fluid. Those values were obtained for the solutions with 5% volume fraction. The results obtained with NFs of rod-shaped particles were compared with the Hamilton and Crosser model [119] and the ones obtained with NFs of spherical shaped particles were compared with the Bruggeman model [72]. The experimental results were, respectively, 12% and 16% higher than that predicted by the theoretical models. Additionally, the increase in thermal conductivity with the increasing volume fraction of NPs was linear in theoretical results, which was not the case for experimental results in small concentrations of NPs. Years later, Murshed et al. [120] proposed two new theoretical models for the calculation of the thermal conductivity of NFs with spherical and cylindrical NPs. The new models were compared to the Maxwell model [71], Hamilton and Crosser model [119], and Prasher model [121] and experimental results. The experimental tests were performed with NFs of TiO_2_ and Al_2_O_3_ NPs in 1 to 5% volume concentrations. Two different base fluids were used, water and EG. Additionally, about 0.1 mM of Cetyl Trimethyl Ammonium Bromide (CTAB) surfactant was added to the NFs. The tests were performed in a temperature range from 20 to 60 °C. The novel models showed better predictions comparing to the experimental results than the classical models.

In 2007, Beck et al. [61] reported a successful correlation between the classical theoretical models Maxwell [71], Hamilton and Crosser [119], and Yu and Choi [122], and the experimental measurements of the thermal conductivity of NFs. The NFs used were dispersions of alumina NPs in EG of 1, 3, and 4 mass fractions, measured at temperatures ranging from, approximately, 25 to 138 °C. The authors reported a better correlation between the results and the models with adjustable parameters, as the case of the shape factor in the Hamilton and Crosser model [119], or the ordered liquid layer thickness in the Yu and Choi model [122]. For instance, the shape factor used was n = 3.4, suggesting that not all the particles remained spherical since some formed agglomerates.

**Figure 17:**
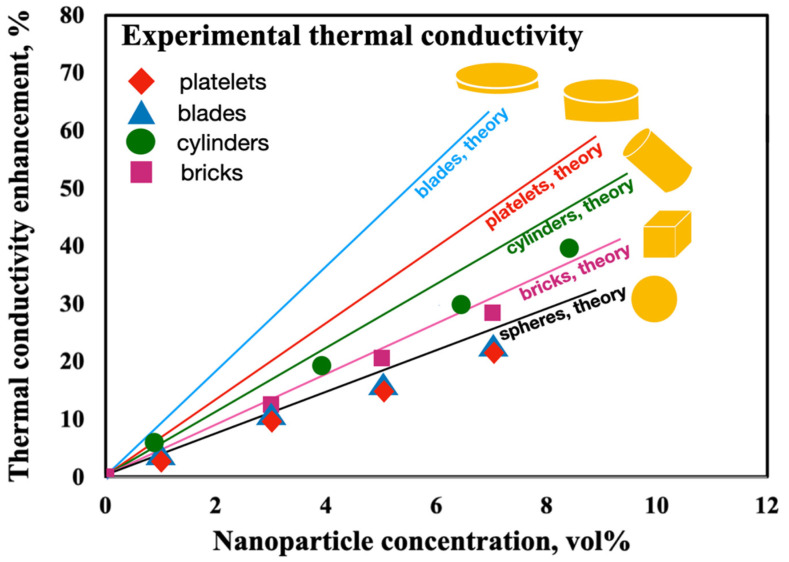
Experimentally measured thermal conductivity of Al_2_O_3_ NFs in EG/Water in function of nanoparticle concentration compared to predictions of H-C model for corresponding particle shapes (adapted from Timofeeva et al. [123]).

In 2009, Timofeeva et al. [123] studied the thermal conductivity of various shapes of alumina NPs in a fluid consisting of equal volumes of EG and water. Experimental results were evaluated by theorical modeling. A synthesis of results can be visualized in Figure 17, where different particles shapes measure at room temperature (21 ± 0.5 °C) are presented as a function of nanoparticle volume fraction. It was possible to observe that thermal conductivity of NFs linearly increases with the increase in nanoparticle volume fraction, for all tested particle concentrations. The researchers [123] used the Hamilton and Crosser equation [119] to estimate thermal conductivity enhancement due to the particle shape. The predictions of model are higher than experimentally measured thermal conductivity, especially for blade and platelet particle shapes.

In 2007, Yoo et al. [44] studied the thermal conductivity of a water based Al_2_O_3_ nanofluid and EG based Fe nanofluid with volume concentrations of 0.3, 0.5, 0.7, 1.0, and 1.5% and 0.2, 0.3, 0.4, 0.55%, respectively. The alumina NFs showed an enhancement of up to 4% and the results agreed to those predicted by the Hamilton and Crosser model [119]. On the other hand, the values obtained experimentally for the iron nanofluid were superior to those obtained theoretically, with an enhancement of up to 18%.

In 2012, Yiamsawasd et al. [49] measured the thermal conductivity of TiO_2_ and Al_2_O_3_ NPs suspended in a water and a 20/80 by mass of EG/water mixture. The fluids had a NPs volume concentration of 0–8% and were tested at temperature of 15–65 °C. Thermal conductivity of NFs was higher than the base fluid and varies with temperature and concentration level of NFs. The results were compared to the predictions obtained by the theoretical models Hamilton and Crosser [119], Bruggeman [72], Yu and Choi [122], and Xie model [62]. The authors reported that the models failed to predict the thermal conductivity ratio in terms of both concentration and temperature of the NFs.

Agarwal et al. [105] using NPs size in the range of 40–55 nm from Fe_2_O_3_, compared the experimental measurements of thermal conductivity NFs against standard theorical model and artificial neural network approach. Estimation using the Maxwell model [71], Bruggeman [72], and the Yu and Choi model [122] exhibited a significant deviation from the experimental results, while the performance of the Hamilton and Crosser model [119] was in the acceptable region. On the other hand, when using artificial neural network approach, predictions were very close to experimental results showing significant learning by establishing concentration and temperature dependence of thermal conductivity [105]. Comparing the predictions using the artificial neural network approach with the Hamilton and Crosser model, average percentage errors were 0.11% and 1.19% for Fe_2_O_3_/water NFs and 0.17% and 1% for Fe_2_O_3_/EG NFs, respectively.

In the same year, Okonkwo et al. [124] compared experimental and theorical methods of obtaining the thermal properties of NFs, among them the thermal conductivity. The authors investigated the thermal performance of Al_2_O_3_ NFs and Al_2_O_3_–Fe hybrid NFs, using water as the base fluid, temperature ranges from 25 to 65 °C and nanoparticle concentration of 0.05%, 0.1%, and 0.2%. The results showed that the Maxwell model [71] greatly overestimate the values of the thermal conductivity, especially at higher volumetric concentrations.

The Bruggeman model [72] presented similar results to the Maxwell model [71] at lower volumetric concentrations, but had far higher values of thermal conductivity at concentrations of 0.2% and the Yu and Choi model [122] showed better results than those obtained experimentally. Okonkwo et al. [124] supposes that this may be because the model is a more dynamic model, responsible for the impact of nanolayers on the thermal conductivity of the particles in the liquid suspension.

For the majority of the studies, the classical models, when compared to the experimental results, failed to predict the increase in thermal conductivity of the NFs. The poor stability of the suspension of NPs in NFs is well known to the scientific community that has been looking for alternatives to get around the problem. The solution has been to use methods, such as adding surfactants, reducing the concentration to delay sedimentation, changing the pH of samples, using magnetic fields, etc. According to Keblinski et al. [125], the particle aggregation and the formation of extended structures of linked NPs may be responsible for much of the disagreement between experimental results and the predictions of the theories.

In recent years, several authors have been exploring the use of computational intelligence to predict the thermal conductivity enhancement of NFs. Alade et al. [126] used support vector regression (SVR) models to predict the thermal conductivity of metallic NFs. The SVR is a computational algorithm developed by Cortes and Vapnik in 1995 [127] that derives from statistical learning theory. Certain data inputs are given to the algorithm that are then mapped via a Gaussian kernel non-linear mapping function onto n-dimensional feature space. The result is an estimation as close as possible to the reference value with a certain precision. The estimation is based on the given training dataset. For that study, 118 and 156 datasets for metallic and metallic oxide-based NFs, respectively, were used. The NFs had water, EG, or transformer oil as base fluid and the NPs were aluminum or copper based. The input data were the suspension temperature, the volume ratio, the particle size, and the thermal conductivities of base fluids and NPs. The authors reported less deviation from the data when using the SVR compared to the Hamilton and Crosser model [119]. The model was also evaluated through statistical parameters. The correlation coefficients were 99.3% and 96.3% and the root mean square error were 1.11 and 1.33 for the metallic and metallic oxide NFs, respectively.

Zhang et al. [128] developed the Gaussian process regression (GPR) model. GPRs are nonparametric kernel-based probabilistic models. Given a certain input, the model will return the estimated value of thermal conductivity enhancement based on the previous databases used to train the model. The inputs used were the thermal conductivity of the base liquid, thermal conductivity of the nanoparticle, the particle size, volume fraction of the nanoparticle, and temperature. The dataset used for training was formed by NFs with metallic and ceramic NPs with water, EG, or transformer oil as base fluids. The authors reported a correlation coefficient of 99.999%, a root mean square error of 0.0030, and a mean absolute error are of 0.0022 for the proposed model.

Khosrojerdi et al. [129] used multilayer perceptron (MLP) artificial neural network (ANN) to predict the thermal conductivity of water based NFs with graphene nanoplatelets. The ANN is formed by several neurons that are placed in different layers. The first layer corresponds to the input layer and the last the output layer. The number of layers and the number of neurons in each layer depends on the weight and the number of neurons of previous layer. The input parameters used were temperature and weight percent of nanofluid and the output the thermal conductivity. The training of the model was performed using data of water-based NFs with graphene nanoplatelets in 0.00025, 0.0005, 0.001, and 0.005 wt.% at temperatures of 25 °C to 50 °C. The index of the root mean square error was 0.04 W/mK, of the correlation coefficient was 99% and of the mean absolute percentage error was 0.26%.

Although theoretical models for predicting the thermal conductivity of NFs confirm many of the values obtained experimentally, at the end of this chapter, the lack of consensus among researchers is evident. Theoretical models serve as a guide for the researcher who can change the composition of his samples according to the answer given by the theoretical calculation instead of using expensive numerical simulations. However, the existing divergences may not be related to the expressions used in the models and they may be being misused by the researchers. This may happen by ignoring, for example, the type or size of the nanoparticles used in the models, or even, performing wrong measurements of the information (properties and characteristics, both of the nanoparticles and the base fluid) that are inserted in the expressions.

## 6. Conclusions

In this review, the most relevant results from the literature regarding the thermal conductivity of NFs have been reported and several controversial results were discussed. Additionally, different techniques used to measure the thermal conductivity of these colloidal mixtures were shown. However, many of them are adaptations from traditional methods frequently used to measure solids, powders, and gases. The main problems of the thermal conductivity devices to measure the NFs, are the influence associated with the convection caused by the fluids and the difficulty to control the temperature where the thermal conductivity tests are performed. In addition, stability, sedimentation, aggregation, and motion of the NPs during the realization of measurements are also problems that are very likely to influence the thermal conductivity results. Hence, several critical issues should receive special attention in order to improve the results obtained from the techniques frequently used to measure the NFs thermal conductivity:

- The calibration of the sensors and/or thermocouples used at different techniques need to be highly accurate;
- The source power needs to generate low and uniform heat flux on the samples to avoid convection;
- The dispersion techniques influence the thermal conductivity and cannot be neglected, such as changes in pH, use of surfactants, among others;
- The methods need to include the motion of NPs, such as Brownian motion and gravitational force, during the treatment and evaluation of the data;
- In some kind of devices, the effects of the sensor power and its depth on the samples must be evaluated in the measurements;
- The effect of the direction of the heat flow imposed on the nanofluid sample (used in some devices), from top to bottom or from bottom to top, has not yet been investigated.

Some possible solutions to improve the reliability and acceptance of these techniques are as follows:

- To minimize the convection effects of the NFs, methods should use samples with low volumes in order to have shortest test times and small temperature variations;
- Before performing thermal conductivity measurements, it is recommended to use techniques to disperse the NPs, such as the sonification process, in order to improve the stability of the NPs suspended in the NFs;
- To increase the number of researchers comparing more than one experimental technique when performing the thermal conductivity measurements;
- The theoretical equations used in the calculations performed by the operator or by the software from equipment needs to be known and considered, as this guarantees the measurement reliability interval.

There is no doubt about the thermal benefits of the NFs, however, the diversity of the thermal conductivity values obtained from different measurement techniques indicates that more studies are required to improve not only the reliability of the results but also the acceptance of these techniques by researchers working in this field. Thus, this comprehensive review showed the advantages and drawbacks of the most used thermal conductivity techniques to measure the NFs, may provide a guidance to researchers interested to implement, improve, and develop the most appropriate experimental protocol to measure the NFs thermal conductivity.

## Figures and Tables

**Table 1:** Advantages and drawbacks of the most used methods to measure the thermal conductivity of NFs.

Methods	Advantages	Drawbacks
Transient hot-wire (THW)	- Measurements are faster (0.1 s to 1 s) [37]; - Small temperature variations are necessary [39].	- Problems from the electrical conducting properties of the fluids [64]; - The contact between the fluid and the wire of the probe/hot-wire can generate secondary path flows of current [64]; - Fluid can polarize or deposit at the surface of the wire [64]; - Dual path conduction can affect the automatic Wheatstone bridge [64].
Steady-state parallel-plate method	- A small volume of the fluid sample are necessary [18]; - The heat transfer it is imposed in one direction [18].	- The temperature increase in each thermocouple needs to be accurately measured [16]; - When the thermocouples are at the same temperature, the difference in temperature readings need to be minimized [16].
Laser flash method (LFM)	- There is no convective heat transport during measurement [68]; - Presents wide range of measurement [97]; - It has high accuracy and repeatability [97]; - Easy sample preparation [97].	- Is not suitable measure NFs with low thermal conductivity [98].
3*ω* method	- Requires small amounts of fluids [12].	- More suitable for non-spherical particles, i.e., more indicate for nanotubes, nanowires, and nanofins [12,88].
Transient plane source (TPS)	- Simultaneously determine the thermal conductivity, thermal diffusivity, and specific heat capacity from a single measurement.	- The convection of the liquids is the biggest problem [82].
Temperature oscillation technique	- Simultaneously measures diffusivity and thermal conductivity of the NFs [22].	- Very dependently of the time period and the amplitude of the temperature oscillation [85].
Coaxial cylinders method	- Good control of heat flux generated [89]; - Very accurate measurement of the heat flow [89].	- Very small temperature gradients are necessary to avoid natural convection [90].
Modified Transient Plane Source (MTPS)	- The shortest test time (0.8 s) [92]; - Minimal sample volume requirement (1.25 mL) [92]; - Low-energy power flux to the specimen under test [92].	- To measure an unknown sample, an iterative method m* is required, described in US Patent 6,676,287 [92].
Extended 3ω method	- Requires only a single droplet of volume size [93].	- It is bad when measuring NFs with less thermal conductivity and heat capacity [93].
Sub-µL Thermal conductivity	- Low volumes samples [24].	- Not possible use it with higher temperatures or volatile base fluids [24].
Steady flow method (SFM)	- The effects of buoyancy and natural convection of the liquids are includes [95].	- Not informed by the authors [95].

## Data Availability

Not applicable.
